# Antibiotic resistance, molecular characterizations, and clinical manifestations of Campylobacteriosis at a military medical center in Hawaii from 2012–2016: a retrospective analysis

**DOI:** 10.1038/s41598-018-29461-z

**Published:** 2018-08-06

**Authors:** Evan C. Ewers, Sarah K. Anisowicz, Tomas M. Ferguson, Scott E. Seronello, Jason C. Barnhill, Michael B. Lustik, Willie Agee, Michael A. Washington, Md A. Nahid, Mark W. Burnett, Ladaporn Bodhidatta, Apichai Srijan, Supaporn Rukasiri, Patcharawalai Wassanarungroj, Sirigade Ruekit, Panida Nobthai, Brett E. Swierczewski, Woradee Lurchachaiwong, Oralak Serichantalergs, Viseth Ngauy

**Affiliations:** 1 Tripler Army Medical Center, Honolulu, HI USA; 2 Walter Reed National Military Medical Center, Bethesda, MD USA; 3 Armed Forces Research Institute of Medical Sciences, Bangkok, Thailand

## Abstract

Hawaii has one of the highest incidences of Campylobacteriosis in the United States, but there remains little published data on circulating strains or antimicrobial resistance. We characterized 110 clinical *Campylobacter* isolates (106 C. *jejuni*, 4 *C*. *coli*) processed at Tripler Army Medical Center in Honolulu, HI from 2012–2016. Twenty-five percent of *C*. *jejuni* isolates exhibited fluoroquinolone (FQ) resistance, compared with 16% for tetracycline (TET), and 0% for macrolides. Two of the four *C*. *coli* isolates were resistant to FQ, TET, and macrolides. *C*. *jejuni* isolates further underwent multilocus sequence typing, pulsed-field gel electrophoresis, and molecular capsular typing. Nineteen capsule types were observed, with two capsule types (HS2 and HS9) being associated with FQ resistance (p < 0.001 and p = 0.006, respectively). HS2 FQ-resistant isolates associated with clonal complex 21, possibly indicating clonal spread in FQ resistance. Macrolides should be considered for treatment of suspect cases due to lack of observed resistance.

## Introduction

Campylobacteriosis, usually caused by *Campylobacter jejuni*, is the most common cause of bacterial gastroenteritis in the United States, responsible for 1.3 million cases annually. It is usually contracted through consumption of contaminated food products – poultry, dairy, pork, or contaminated water^1^. While primarily a self-limited infection, serious complications including: colitis, cholecystitis, bacteremia, meningitis, reactive arthritis, irritable bowel syndrome, and Guillain-Barre syndrome (GBS) have been reported^2–5^. Recent FoodNet data showed 20% of cases result in hospitalization, while 0.3% of infections were fatal, highlighting the potential morbidity and mortality associated with Campylobacteriosis^6^.

The state of Hawaii has one of the highest incidences of *Campylobacter* infection in the nation for the past three decades, ranging from 3–6 times the national average, most recently 36.19 versus 11.79 per 100,000 persons, respectively^7–9^. The cause of Hawaii’s higher incidence of Campylobacteriosis is unknown; however, locally-sustained infection over sporadic outbreaks is a proposed explanation^8,10^. An ecological study of select pathogens on the more densely populated island of O’ahu identified *Campylobacter* species in 18/22 freshwater streams discharging near recreational beaches in both urban and agricultural areas, suggesting environmental sources could contribute to the disease burden in Hawaii^11^.

Recently, antibiotic-resistant *Campylobacter* was labeled a pathogen of “serious” concern by the Centers for Disease Control and Prevention (CDC) as well as a “high” priority pathogen for the development of new antimicrobial agents by the World Health Organization. Most concerning is the rising resistance to fluoroquinolones (FQ) and macrolides^12,13^. In the United States, the rate of FQ resistance has increased over the past several years from 21.6% in 2012 to 26.7% in 2014^14^. Globally, the highest rates of FQ resistance are seen in Southern and Eastern Asia, and have been increasing over the past several decades, including in returning Western travelers^12,15–19^. In contrast, macrolide resistance has remained low (<10%), arguing for their use as empiric traveler’s diarrhea treatment for patients traveling to or returning from South and Southeast Asia^20^.

Ongoing efforts to develop a *C*. *jejuni* vaccine have focused on developing a capsular polysaccharide conjugate vaccine. However, a lack of information on circulating capsular types remains a significant limitation to vaccine development^21^. Pike *et al*. published a systematic review on the epidemiology of *C*. *jejuni* capsular types, noting the vast majority of information on circulating strains came from Europe (87%), with the US, Asia, and Oceania making up 12%, highlighting the need for more global serotypes to guide multivalent vaccine development^22^.

Despite the high incidence of *Campylobacter* infection in Hawaii, there is little data regarding the circulating strains, antimicrobial resistance, or risk factors associated with its acquisition. In this study, we sought to characterize Campylobacteriosis in Department of Defense (DoD) and Veteran’s Affairs (VA) beneficiaries in Hawaii through antimicrobial sensitivity testing (AST), genetic typing, molecular capsular typing, and clinical presentation to guide the use of antibiotics for empiric treatment of active infections in residents of and travelers to Hawaii, with the goal of guiding clinical decisions for empiric antibiotic choice in treating active *Campylobacter* infections, as well as provide insight for future capsular vaccine development.

## Methods

### Study Population and Clinical Chart Reviews

This protocol was approved by the Tripler Army Medical Center (TAMC) Institutional Review Board (Protocol #15R28). TAMC is a tertiary care referral hospital in Honolulu, HI serving DoD and VA beneficiaries in the Asia-Pacific. Informed consent was waived by the TAMC Institutional Review Board, and all research was conducted in accordance with appropriate guidelines and regulations. Stored *Campylobacter* isolates collected from stool samples submitted for evaluation of symptomatic diarrheal disease were included in this study. The beneficiary population in Hawaii is well-integrated into the local economy, participating in similar activities and consuming from the same food sources as the local population.

Retrospective chart reviews were performed on the laboratory-confirmed cases. Epidemiological data included age, sex, recent antibiotic use (within 3 months), occupation, potential food exposures, travel history, and pets. Pediatric patients were divided into two groups: 0–8 years and 9–18 years due to side effect concerns with specific antibiotic classes (tetracyclines and fluoroquinolones). Adults were subgrouped by decade until 40 years of age since these groups reflect different populations seen at our facility: young active duty military and deployment populations, longer-serving military populations, and retiree and veteran populations. We reviewed clinical data, including temperature, vomiting, diarrhea, bloody stool, fecal leukocytes, white blood cell count, serum chemical parameters (liver-associated enzymes, serum creatinine), treatment offered, and response to therapy.

### Clinical Samples, Microbiology, and Antimicrobial Sensitivity Testing

One hundred ten *Campylobacter* isolates obtained from January 2012 – February 2016 were shipped to the Armed Forces Research Institute of Medical Sciences (AFRIMS) in Bangkok, Thailand using Microbank^TM^ cryobeads (Prolab Diagnostic, Round Rock, TX). Each sample was inoculated into Preston selective enrichment broth and incubated under microaerobic conditions (37 °C, 10% CO_2_, 5% O_2_ and 85% N_2_) for 24 hours. Subsequently, the isolates were sub-cultured on Brucella agar plate with 5% sheep blood and incubated at 37 °C under microaerobic conditions. After 48–72 hours, isolate identification was confirmed using motility examination and phenotypic testing including oxidase, catalase, indoxyl acetate hydrolysis, rapid hippurate hydrolysis, nitrate reduction, growth temperature and oxygen tolerance tests^23^. As part of an investigation into emerging culture-independent microbial identification methods, a subset of isolates (n = 49) were also analyzed using polymerase chain reaction/electrospray ionization mass spectrometry. This method found that all tested isolates could be identified to the genus level and were consistent with the conventional identification and molecular testing performed at AFRIMS (see Supplementary Information, Supplementary Table S1).

Antimicrobial susceptibility testing to azithromycin (AZM), erythromycin (ERY), nalidixic acid (NAL), ciprofloxacin (CIP), tetracycline (TET) and ceftriaxone (CRO) was performed on confirmed isolates using commercially available E-tests (Liofilchem, Roseto degli Abruzzi TE, Italy) to determine the minimal inhibitory concentration (MIC). The latter was used as an internal control since *C*. *jejuni* is resistant to cefoperazone. Susceptibility results were interpreted following Clinical and Laboratory Standards Institute (CLSI) guidelines and National Antimicrobial Resistance Monitoring System (NARMS) using *Campylobacter jejuni* ATCC 33560 as the control strain^24^. The CLSI guidelines do not offer susceptibility recommendations for *Campylobacter* to CRO, so were interpreted following the guidelines of *Enterobacteriaceae*^25^.

### Capsular Typing

Capsule typing was performed on genomic DNA extracts of *C*. *jejuni* isolates with four multiplex primer sets using 36 specific primers targeting capsule genes as developed and PCR reactions developed by Poly *et al*.^26,27^. Two µL of each *C*. *jejuni* isolate DNA was subjected to each multiplex PCR in a 25 µL reaction mixture containing 1X PCR buffer (10 mM Tris–HCl, pH 8.3, 50 mM KCl), 2.0 mM MgCl2, 300 µM concentration of each dNTPs (deoxynucleotide triphosphate), 0.4 µM of each primers sets (Alpha, Beta, Gamma and Delta) and 2.5 U of AmpliTaq Gold DNA polymerase. Amplification steps were as follows: 94 °C for 5 min; 28 cycles of 94 °C for 1 min, 52 °C for 1 min and 72 °C for 1 min and a final extension step at 72 °C for 10 min. Amplicons were visualized after gel electrophoresis at 120 V for 1 hour on a 2.0% Agarose-1000 gel (Invitrogen, USA) for Beta and Gamma set and 2.5% Agarose-1000 gel for Alpha and Delta set and staining with ethidium bromide. DNA *of C*. *jejuni* of known capsule types and 2-log DNA ladder (New England BioLabs, USA) were used as positive controls and a size marker, respectively.

### Multilocus Sequence Typing (MLST)

MLST was performed according to developed protocol on seven housekeeping genes (protein product shown in parentheses): *aspA* (aspartase A), *glnA* (glutamine synthase), *gltA* (citrate synthase), *glyA* (serinehydroxymethyl transferase), *pgm* (phosphoglucomutase), *tkt* (transketolase), and *uncA* (ATP synthase-α)^28^. Sequences-based identification of MLST profiles used Bionumerics Version 7.5 with the MLST plugin (Applied Maths NV, Belgium). Isolates were characterized by their sequence type (ST) and as members of clonal complexes (CC). The MLST profiles identified in this study were submitted to the PubMLST database http://pubmlst.org/campylobacter^29^.

### Pulsed Field Gel Electrophoresis (PFGE)

PFGE of *C*. *jejuni* using *SmaI* was performed according to standard protocol^30^. The agarose DNA plug was digested with 40 U *SmaI* (Roche, Germany) according to the manufacturer’s instructions. PFGE was performed in a CHEF Mapper system (Bio-Rad, USA) at 14 °C in 0.5x TBE (Tris/borate/EDTA). Run times and pulsed times were 6.76–35.38 s for 18 h with linear ramping. Gels were stained with ethidium bromide (0.5 µg/ml), and band patterns were visualized by Gel Documentation System (Syngene, United Kingdom). Gel images were analyzed with BioNumerics version 7.5 (Applied Maths, Belgium) to obtain a phylogenetic tree. Cluster analysis of the Dice similarity indices based on UPGMA was done to generate a dendrogram describing the relationship among each *C*. *jejuni* isolate.

### Statistical analysis

Chi-square tests and Fisher’s exact tests were done to assess categorical associations between variables. All statistical analyses were conducted using SAS version 9.2 (SAS Institute, Cary, NC).

### Data availability

The datasets generated during and/or analyzed during the current study are available from the corresponding author on reasonable request.

### Disclaimer

The views expressed in this article by the authors do not represent those of the United States Army, the Department of Defense, or the United States Government.

## Results

### Clinical Data

A retrospective chart review of 110 patients with positive cultures was performed to evaluate the clinical presentation and potential risk factors for Campylobacteriosis in Hawaii. Species was identified as *C*. *jejuni* in 106 isolates and *C*. *coli* in 4 isolates. The demographic breakdown of cases can be seen in Table 1. Seasonality of infection was observed: 61% of *C*. *jejuni* isolates were collected between January and June compared with 39% from July through December; over half (53%) were collected between May and August, suggesting a relative summer peak. A travel history was obtained in 69 patients (65 *C*. *jejuni* and 4 *C*. *coli*), and 29% (n = 20) reported international travel within 3 months prior to presentation. All four *C*. *coli* cases reported recent international travel, compared with only 23% of *C*. *jejuni* cases who reported a travel history (p = 0.005).

**Table 1:** Demographic Data of Campylobacteriosis cases at Tripler Army Medical Center from 2012–2016.

	No. of Cases	(%)
Gender		
Female	48	44
Male	62	56
Age		
0–8	21	19
9–18	11	10
19–29	35	32
30–39	21	19
40+	22	20
Mean (STD) (years)	26.3 (16.6)	
Median (IQR((years)	27 (11–35)	
Campylobacter sp.		
*C*. *jejuni*	106	96
*C*. *coli*	4	4
International Travel^*^	19	28
Year		
2012	22	20
2013	41	38
2014	29	26
2015	13	12
2016	5	5

^*^Total cases = 110. International travel history recorded in 69 patients.

Comparisons regarding common clinical signs and symptoms can be seen in Table 2. Patients ≤8 years were more likely to present with hematochezia (90% vs 48%, p < 0.001), but were less likely to present with abdominal pain or cramping (82% vs. 99%, p = 0.014). Additional laboratory data such as serum chemistries were collected in 56% of patients (n = 62). No patients had liver-associated enzyme elevations greater than twice the upper limit of normal, and only 8% (n = 5) presented with elevated serum creatinine >1.25 mg/dL. Fecal leukocytes were present in 37% of the 48 patients tested. Twenty-two patients (22%) had documented antimicrobial use in the three months preceding infection: beta-lactams (n = 7), fluoroquinolones (n = 3), tetracyclines (n = 4), clindamycin (n = 2), nitrofurantoin (n = 1), trimethoprim-sulfamethoxazole (n = 2), anti-fungals (n = 1). TET resistant isolates were detected in only two patients with a recent history of antibiotic use.

**Table 2:** Key Clinical Presentations of Campylobacteriosis in Hawaii.

	Clinical Metric											
	Gross Blood			Abdominal Pain			Fever^§^			Leukocytosis^¶^		
	No. of Cases^*^	(%)	*P* ^†^	No. of Cases	(%)	*P*	No. of Cases	(%)	*P*	No. of Cases	(%)	*P*
Gender			0.321			1.000			0.419			0.464
Female	28/45	62		42/44	96		19/47	40		12/31	39	
Male	30/58	52		55/57	96		19/59	32		14/48	29	
Age			0.002			0.033			0.083			0.079
0–8	18/20	90		14/17	82		11/21	52		3/8	38	
9–18	7/11	64		11/11	100		4/11	36		5/8	63	
19–29	18/34	53		34/35	97		12/34	35		10/27	37	
30–39	10/19	53		19/19	100		9/21	43		6/16	38	
40+	5/19	26		19/19	100		2/19	11		2/20	10	
Campylobacter sp.			0.316			1.000			1.000			1.000
*C*. *jejuni*	57/99	58		93/97	96		37/103	36		25/75	33	
*C*. *coli*	1/4	25		4/4	100		1/3	33		1/4	25	
International Travel			0.420			0.496			0.569			0.101
No	30/49	61		46/47	98		19/49	39		15/37	41	
Yes	9/18	50		18/19	95		5/17	29		2/15	13	

*These are presented as a fraction of the total number available for given clinical or historical information.

^†^P-values in this table calculated using a two-sided Fisher’s Exact Test with significant values defined as p < 0.05.

^§^Fever define as temperature >100.4°F (>38 °C).

^¶^Leukocytosis defined as >10.4 Giga/L cells.

Overall, 62% of patients received antibiotic treatment (53% of ages 0–18, 65% aged 19+) of patients. Patients ≤18 years were twice as likely to be treated with azithromycin compared with those 19 years or older (44% vs. 22%, p = 0.034). Few complications were seen in our patient population: Four developed colitis, one patient had appendicitis, and one patient had biliary dyskinesia. There was one case of post-infectious irritable bowel syndrome. No cases of GBS were observed. Ancillary historical data including occupation, pet/animal exposure, or specific food exposures were too seldom documented to provide reasonable interpretation.

### Antimicrobial Susceptibility Testing

All isolates underwent AST using an E-test method (Table 3). In total, 26% (n = 29) of our *Campylobacter* isolates were FQ-resistant, and 17% (n = 19) of isolates were TET-resistant (8% resistant to both). All isolates had minimal inhibitory concentrations ≥6 µg/ml to CRO (Supplementary Table S2). When broken down by species, 25% (n = 27) of *C*. *jejuni* isolates were FQ-resistant, but only 16% (n = 17) isolates were resistant to TET. Eight (7.5%) of *C*. *jejuni* isolates were resistant to both CIP and TET. No *C*. *jejuni* isolates were resistant to AZM or ERY. Two (50%) *C*. *coli* isolates from patients with documented travel to Southeast Asia (Cambodia and Philippines) were resistant to the FQs, tetracyclines and macrolides. Overall, international travel was associated with NAL and CIP resistance (53% vs. 20% for those with and without travel history, p = 0.016) and TET resistance (58% vs. 10%, p < 0.001, respectively).

**Table 3:** Antimicrobial Resistance Rates for Campylobacter Isolates Broken Down by Species and Capsule Type.

	No. of Isolates *N*	ST-CC	NAL (%)	CIP (%)	TET (%)	AZM (%)	ERY (%)
*C*. *jejuni*	106		25	25	16	0	0
HS1	1	ST-353	0	0	100	0	0
HS12	5	ST-45	0	0	0	0	0
HS15	3	singletons	67	67	67	0	0
HS5/31, HS15	1	ST-354	0	0	100	0	0
HS19	2	ST-22	0	0	0	0	0
HS2	20	ST-21, singletons	80	80	15	0	0
HS3	7	ST-353, singletons	14	14	0	0	0
HS37	2	ST-443	0	0	0	0	0
HS4-A	10	ST-508	0	0	0	0	0
HS4-AB	23	ST-48, ST-607, singletons	9	9	4	0	0
HS41	1	singleton	0	0	0	0	0
HS42	1	ST-45	0	0	0	0	0
HS44	2	ST-206	0	0	0	0	0
HS5/31, HS45^*^	5	ST-52, ST-574	20	20	20	0	0
HS53	2	ST-354, ST-35	50	50	0	0	0
HS55	2	ST-45	0	0	100	0	0
HS6/7	2	ST-45	0	0	0	0	0
HS8/17	8	ST-21, ST-1287	13	13	25	0	0
HS9	4	ST-45, singletons	75	75	75	0	0
Untypable	5	ST-21, ST-177, ST-353	0	0	20	0	0
*C*. *coli*	4		50	50	50	50	50

Abbreviations: ST-CC = MLST clonal complex, NAL = nalidixic acid, CIP = ciprofloxacin, TET = tetracycline, AZM = azithromycin, ERY = erythromycin.

*These indicated capsule types are both members of the HS5/31-complex^26^.

Minimum inhibitory concentration ranges can be seen in Supplementary Table 2.

### Genotypic and Capsular Typing

**Figure 1:**
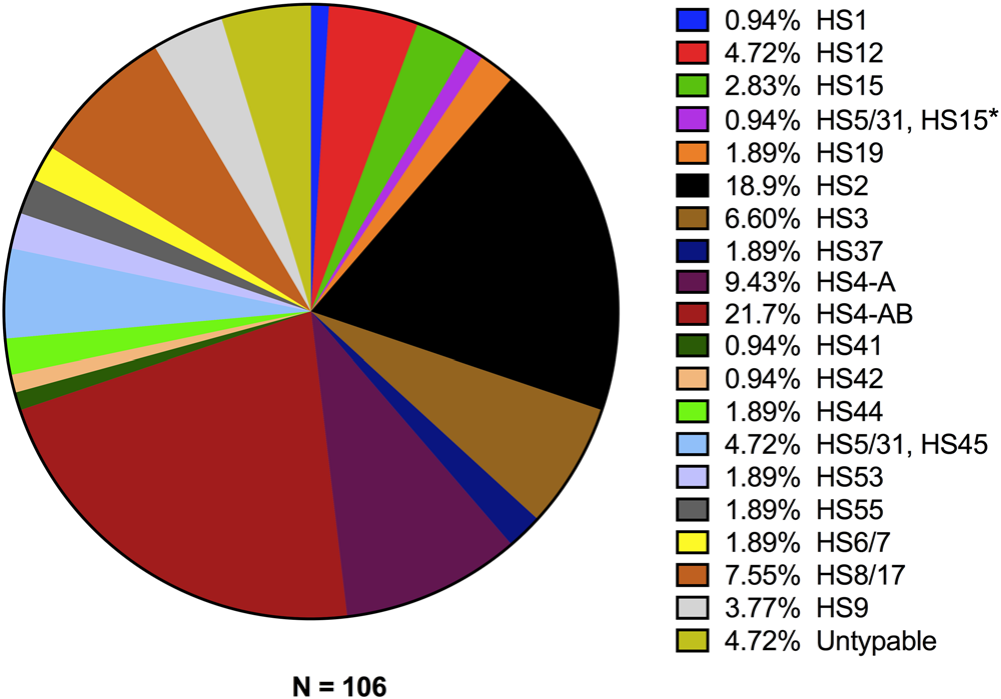
Percentage of *C*. *jejuni* (N = 106) isolate capsular types as determined by multiplex PCR. HS5/31, HS15 and HS5/31, HS45 isolates are both part of the HS5/31-complex.

*C*. *jejuni* capsule typing using a multiplex-PCR method, PFGE and MLST were performed to better describe circulating *C*. *jejuni* strains and determine genetic relatedness. Nineteen *C*. *jejuni* capsule types or complexes were identified, and only five isolates were nontypable using the multiplex PCR method (Fig. 1). The most common capsule types were HS2, HS4-A, and HS4-AB, accounting for half the *C*. *jejuni* isolates. Only two capsule types, HS2 and HS9, were associated with increased antibiotic resistance. The HS2 isolates were strongly associated with resistance to NAL and CIP, with 80% (16/20) demonstrating resistance, compared to 13% of other capsule types (11/86) (p < 0.001). HS2 capsular type was not associated with TET-resistance. The HS9 isolates demonstrated a 75% (3/4) resistance rate for CIP and TET. These rates were significantly higher when compared with isolates other than HS2 for CIP (10%, p = 0.006) and with all other isolates for TET (14%, p = 0.013). All four patients with HS9 isolates had documented travel to East Asia or Southeast Asia within 3 months of their infection. Of the available historical data, other capsule types were also seen in returning travelers: HS15 (n = 3, Indonesia, Philippines, Japan), HS5/31,HS15 (n = 1, Korea). Only 1 HS2 isolate (#5) with a documented travel history (n = 15) was associated with recent international travel (Philippines).

**Figure 2:**
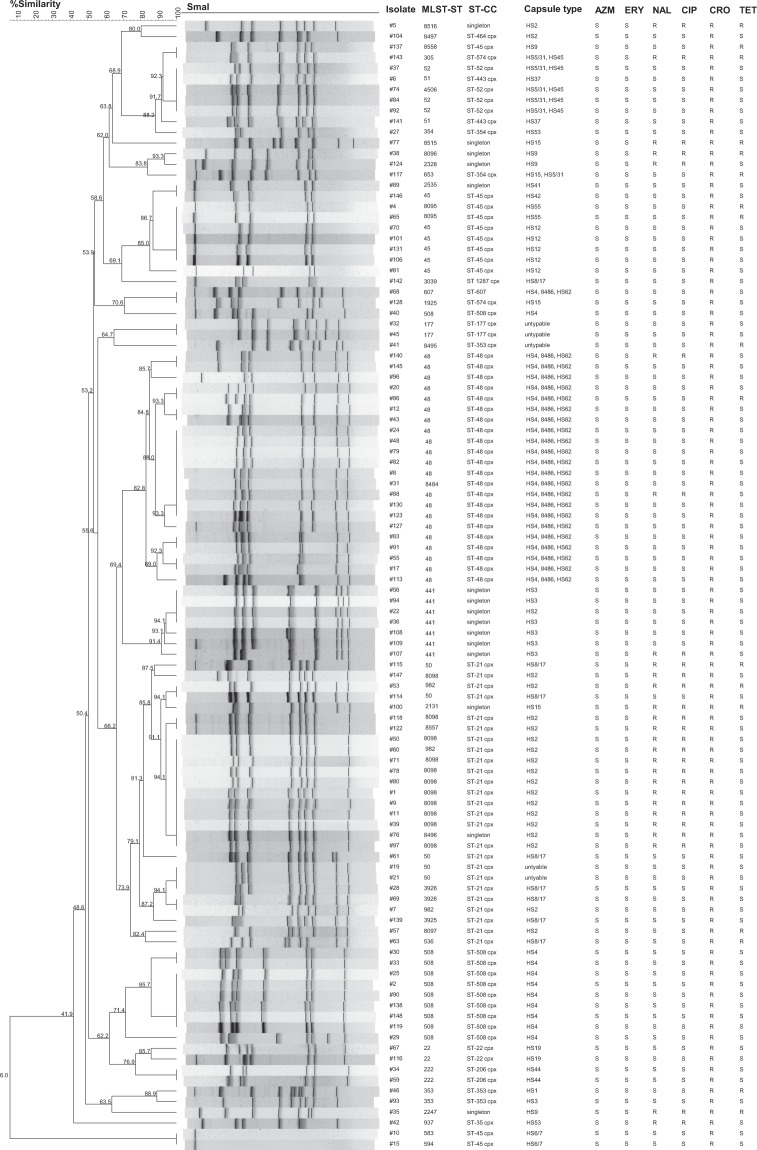
Dendogram of 106 *C*. *jejuni* Isolates with MLST sequence types and clonal complexes, and antimicrobial susceptibility patterns. PFGE cluster analysis based on *SmaI* banding patterns. Bootstrap values indicate % similarity. At 80% similarity, there are 23 different genotypes. The two largest clades are made up of HS2 and HS4-AB isolates, which show high-degrees of clonality. Abbreviations: MLST-ST– multi-locus sequence typing-sequence type; MLST-CC – multi-locus sequence typing-clonal complex; AZM – azithromycin; ERY – erythromycin; NAL – nalidixic acid; CIP – ciprofloxacin; CRO – ceftriaxone; TET – tetracycline.

A *smaI* dendogram using all 106 *C*. *jejuni* isolates was developed based on their PFGE genotypes (Fig. 2). At an 80% similarity, there were 23 different genotypes observed. MLST revealed 41 different STs, of which 12 were new. These new STs accounted for 22% (n = 23) of the *C*. *jejuni* isolates, and ST-8098 predominated, comprising 11 of the 23. Half of the 12 new STs contained new alleles, while the other half was new combinations of previously-assigned alleles. Out of the 41 STs, 32 belonged to 16 clonal complexes (CC), and nine were singletons, not belonging to any recognized CC. The most common PFGE genotypes were dominated by three CC: ST-48, ST-21, ST-508 complexes; associated with capsular types HS4-AB, HS2 and HS 8/17, and HS4-A, respectively. The FQ-resistant HS2 isolates showed a high-degree of genetic similarity, with 11/16 of the isolates demonstrating identical genotypes. The only CIP-resistant HS2 isolates that were not members of CC-21 were singletons (#5 and #76). In comparison, the three HS9 isolates that were also FQ-resistant did not belong to any CC and were singletons with genetic diversity by PFGE as well, with 2 different genotypes at an 80% similarity level. With one exception (#68), the HS4-AB isolates demonstrated >80% similarity. There was no genotypic clustering for TET-resistant isolates.

## Discussion

The clinical presentations seen in this study are consistent with previously published studies on *Campylobacter* infection in pediatric and adult populations^3,8,31^. HS1/44 and HS4-AB capsular types have recently been associated with the development of GBS, however, no patients in our study developed GBS or polyneuropathy^32^. Our sample size is likely too small to detect the very small incidence of GBS (0.07%)^5^. We noted a higher rate (20%) of recent antibiotic use within the period three months prior to the incident infection compared to 9% in the 28 days prior reported by Effler and colleagues^10^. Although we evaluated recent antibiotic use over a longer preceding time period, our study supports the association between an increased risk of *Campylobacter* infection with recent antibiotic use.

NARMS data from 2014 noted US mainland CIP-resistance rates of 26.7% for *C*. *jejuni* and 35.6% for *C*. *coli* in clinical isolates^14^. The NAL and CIP-resistance rate of 25% seen in our study is comparable to that of the US mainland (p = 1.00). In contrast, the rate of TET resistance in *C*. *jejuni* isolates was significantly lower than that reported in NARMS (16% vs. 48.6%, p < 0.001). The lower TET-resistance observed in our study is somewhat surprising considering the widespread use of tetracyclines as livestock growth promoters, and the high resistance rates reported in isolates from the both the US mainland and Asia. Hawaii currently follows the Food and Drug Administration guidance on the use of antibiotics in livestock, permitting the use of tetracyclines. The *C*. *coli* isolates in our study had a higher proportion that were antibiotic-resistant when compared with *C*. *jejuni* isolates, which has been demonstrated in human and agricultural samples, and particularly in relation to macrolides^14,16,33,34^. The resistance rates seen in our population appear to more closely resemble the US mainland than the higher-reported rates seen in Asia. Unfortunately, there is no data for comparison on antimicrobial resistance in *Campylobacter* species in the broader Pacific islands.

Globally, the two most common reported capsular types in the literature are HS4/HS4-complexes and HS2. In their systematic review of the global distribution of *C*. *jejuni*, Pike *et al*. reported that of the available data, HS4-complexes comprised 23.5% of isolates in North America, while HS2 accounted for 10.7%. In contrast, HS2 capsular types were more common in Asia (11%) than HS4-complexes (8.9%)^22^. In our study, HS2 capsule types comprised 18.2% of our *C*. *jejuni* isolates and were strongly associated with FQ-resistance suggesting a potential for the development of increasing FQ-resistance in *C*. *jejuni* strains in Hawaii. The majority of HS2 isolates (ST-8098) were grouped under CC-21, which has been isolated from multiple different sources including humans, chickens, cattle, and the environment. In two recent studies, Kovac and colleagues demonstrated clonal spread of CC-21 *C*. *jejuni* isolates from multiple sources in Europe that harbored FQ resistance^35,36^. Unfortunately, *C*. *jejuni* capsule typing was not performed as part of their investigations. The demonstrated clonal spread arising from multiple sources and the high rate of FQ resistance makes HS2/CC-21 isolates of particular concern in Hawaii and emphasize the necessity of continued surveillance.

The polysaccharide capsule is a major determinant in *C*. *jejuni* immunogenicity and pathogens, and has been proposed as a target for vaccine development. Capsule-type predominance differs geographically; however, the HS4-complexes, HS2, and HS1/44 are most commonly identified. Capsular-typing methods utilizing the Penner agglutination assay are expensive and complex thus limiting its widespread use. Use of the newer and relatively easier multiplex-PCR assay allowed us to identify a higher proportion of HS2 isolates, HS4 isolates, and HS 8/17 capsule types than would have been anticipated based on previously reported data^22^. The leading *C*. *jejuni* candidate vaccine platform in development undergoing early clinical trials covers the HS23/36 and HS4-complex serotypes^37,38^. The high rate of FQ resistance in HS2 and HS9 capsule types seen in our study suggests these capsular types may need to be integrated into vaccine portfolios.

Although this is the most in-depth assessment of Campylobacteriosis in Hawaii to date, this study has several limitations. Firstly, this was a retrospective study of pre-identified samples positive for Campylobacter infection so no comparator control group was available. Secondly, the clinical information obtained on chart review was highly variable and limited by provider documentation in the medical records and the work up performed. Thirdly, the sample size was relatively small and was taken from a defined population of DoD and VA beneficiaries who commonly are living and working on the island for varying periods of time (weeks to years). While our study did not assess the local population, our patients were generally integrated into the community, engaging in the similar recreational activities and consuming from the same local food source.

While the rate of FQ-resistance rate in *Campylobacter* species in Hawaii is the same as the US mainland, we identified a clonal HS2/CC-21 strain with a markedly higher resistance rate than other circulating strains. With antimicrobial selection pressure, it is possible this strain will continue to propagate in the future, resulting in increased FQ resistance in Hawaii. Our study highlights the need for continued surveillance of the epidemiology and antimicrobial sensitivities of *Campylobacter* species in Hawaii to guide clinical treatment and to inform future vaccine candidate platforms. Due to low resistance rates, macrolides should be considered for empiric treatment of suspected Campylobacteriosis cases in Hawaii.

## Electronic supplementary material

Supplementary Information
