# Supplementary material for: Antibiotic resistance, molecular characterizations, and clinical manifestations of Campylobacteriosis at a military medical center in Hawaii from 2012–2016: a retrospective analysis
Source: Sci Rep. 2018 Aug 6;8:11736. doi: 10.1038/s41598-018-29461-z (PMC6078982; doi:10.1038/s41598-018-29461-z)
Supplement: Supplementary file 1 — Supplementary Information [file 41598_2018_29461_MOESM1_ESM.pdf]

Antibiotic resistance, molecular characterizations, and clinical manifestations of Campylobacteriosis at a military medical center in Hawaii from 2012-2016: a retrospective analysis.

Evan C. Ewers[1,2], Sarah K. Anisowicz[1], Tomas M. Ferguson[1], Scott E. Seronello[1], Jason C. Barnhill[1], Michael B. Lustik[1], Willie Agee III[1], Michael A. Washington[1], Md A. Nahid[1], Mark W. Burnett[1], Ladaporn Bodhidatta[3], Apichai Srijan[3], Supaporn Rukasiri[3], Patcharawalai Wassanarungroj[3], Sirigade Ruekit[3], Panida Nobthai[3], Brett E. Swierczewski[3], Woradee Lurchachaiwong[3], Oralak Serischantalergs[3], and Viseth Ngauy[1]

Affiliations:

1. Tripler Army Medical Center, Honolulu, HI, USA
2. Walter Reed National Military Medical Center, Bethesda, MD, USA
3. Armed Forces Research Institute of Medical Sciences, Bangkok, Thailand.

Send Correspondence to evan.c.ewers.mil@mail.mil.

SUPPLEMENTARY METHODS

Polymerase Chain Reaction/Electrospray Ionization Mass Spectrometry (PLEX-ID) Methods

Genus level identification was performed using the PLEX-ID system by Ibis Biosciences, Abbott. Briefly, the base compositions of specific regions of various ribosomal DNA genes were interrogated by a combination of PCR and mass spectrometry. After incubation and growth, each isolate was removed from the culture plate using a sterile inoculation loop and used to inoculate 500ul of phosphate buffered saline. Isolate was added to the saline until a turbid mixture was produced. Nucleic acid from approximately 300µl of each specimen was extracted using the QIAamp DNA and RNA Mini (Qiagen, Valencia, CA) method and the resulting extract was suspended in 260µl of Qiagen elution buffer AE in accordance with the manufacturer's instructions. Eluted nucleic acids were stored at 4°C until further analysis could be carried out with the PLEX-ID system. Analysis was initiated by the transfer of nucleic acids to the Abbott Broad Bacterial Low Plate (Ibis Biosciences, Abbott, Abbott Park, IL) followed by PCR and mass spectrometry analysis using the PLEX-ID system per a manufacturer developed protocol. Each plate was sent to Ibis Bioscience for mass spectrometry analysis and data interpretation. Base composition data was electronically forwarded to investigators at TAMC by Ibis Biosciences, Abbott staff members. Base composition data was then electronically compared to a proprietary database for genus and determination. This database was maintained and curated by Ibis Biosciences and contained 23 *Campylobacter* strains representing 8 species with base composition signatures defined across 4 to 5 loci.

Table S1: Primer pairs and molecular targets utilized to confirm the genus level identification of 50 of the *Campylobacter* isolates evaluated in this study. All primer pairs targeted ribosomal DNA. The number of adenines (A), guanosines (G), cytosines (C), and thymines (T) amplified by each primer pair was compared to a proprietary database developed by Ibis Biosciences to identify the closest matching genus and species. The 49 isolates evaluated in this study correspond to the *C. jenuni/C. coli* group of *Campylobacter* and are distinct from the *Acrobacter* and *Helicobacter* genera. These results are consistent with the biochemical methods used for bacterial identification at AFRIMS and TAMC.

Primer Pair	Sequence	Molecular Target	Base Count	Organism Match in Ibis database
346	FORWARD TAGAACACCGATGGCGAAGGC REVERSE TCGTGGACTACCAGGTATCTA	16s rDNA	A30 G31 C23 T15	*C. jejuni/C. coli*
348	FORWARD TTTCGATGCAACGCGAAGAACCT REVERSE TACGAGCTGACGACAGCCATG	16s rDNA	A31 G32 C25 T32	*C. jejuni/C. coli*
361	FORWARD TTTAAGTCCCGCAACGAGCGCAA REVERSE TTGACGTCATCCCCACCTTCCTC	23s rDNA	A29 G31 C25 T23	*C. jejuni/C. coli*
349	FORWARD TCTGACACCTGCCCGGTGC REVERSE TGACCGTTATAGTTACGGCC	16s rDNA	A25 G29 C24 T23	*C. jejuni/C. coli*
3350	FORWARD TCCACACGGTGGTGGTGAAGG REVERSE TCCAAGCGCAGGTTTACCCCATGG	50s rDNA	A21 G22 C18 T15	*C. jejuni/C. coli*

RESULTS

Table S2: Minimal inhibitory concentrations (MIC) of tested antibiotics for all *Campylobacter* isolates analyzed in this study. MICs are presented in µg/mL.

Antibiotic	N	Median	Interquartile Range	Minimum	Maximum	Number >max	%>max
Azithromycin	110	0.047	0.032 - 0.064	0.016	>256	2	2
Erythromycin	110	0.25	0.19 - 0.5	0.064	>256	2	2
Nalidixic acid	110	3	2 - >256	1.5	>256	29	26
Ciprofloxacin	110	0.094	0.094 - >32	0.032	>32	29	26
Tetracycline	110	0.032	0.023 - 0.064	0.016	>256	8	7
Ceftriaxone	110	>32	>32 - >32	6	>32	108	97

REFERENCES

1. Farrell JJ, Hujer AM, Sampath R, Bonomo RA. Salvage microbiology: opportunities and challenges in the detection of bacterial pathogens following initiation of antimicrobial treatment. Expert Rev Mol Diagn 2015; 15:349-360.

2. Jacob D, Sauer U, Housley R, Washington C, Sannes-Lowery K, Ecker DJ, et al. Rapid and high-throughput detection of highly pathogenic bacteria by Ibis PLEX-ID technology. PLoS One 2012; 7:e39928.

3. Bissonnette L, Bergeron MG. Multiparametric technologies for the diagnosis of syndromic infections. Clin Microbiol Newsl 2012; 34:159-68.
